# Supplementary material for: A safety cap protects hydrogenase from oxygen attack
Source: Nat Commun. 2021 Feb 2;12:756. doi: 10.1038/s41467-020-20861-2 (PMC7854748; doi:10.1038/s41467-020-20861-2)
Supplement: Supplementary file 1 — Supplementary Information [file 41467_2020_20861_MOESM1_ESM.docx]

Supplementary Information

**A safety cap protects hydrogenase from oxygen attack**

Martin Winkler, Jifu Duan, Andreas Rutz, Christina Felbek, Lisa Scholtysek, Oliver Lampret, Jan Jaenecke, Ulf-Peter Apfel, Gianfranco Gilardi, Francesca Valetti, Vincent Fourmond, Eckhard Hofmann, Christophe Léger*, Thomas Happe*

*Correspondence to:  [thomas.happe@rub.de](mailto:thomas.happe@rub.de)

christophe.leger@imm.cnrs.fr

**This PDF file includes:**

- - Supplementary Figures 1 to 17
  - Supplementary Tables 1-3
  - Supplementary Notes 1-2
  - Supplementary Discussion 1-2

Supplementary Figures:


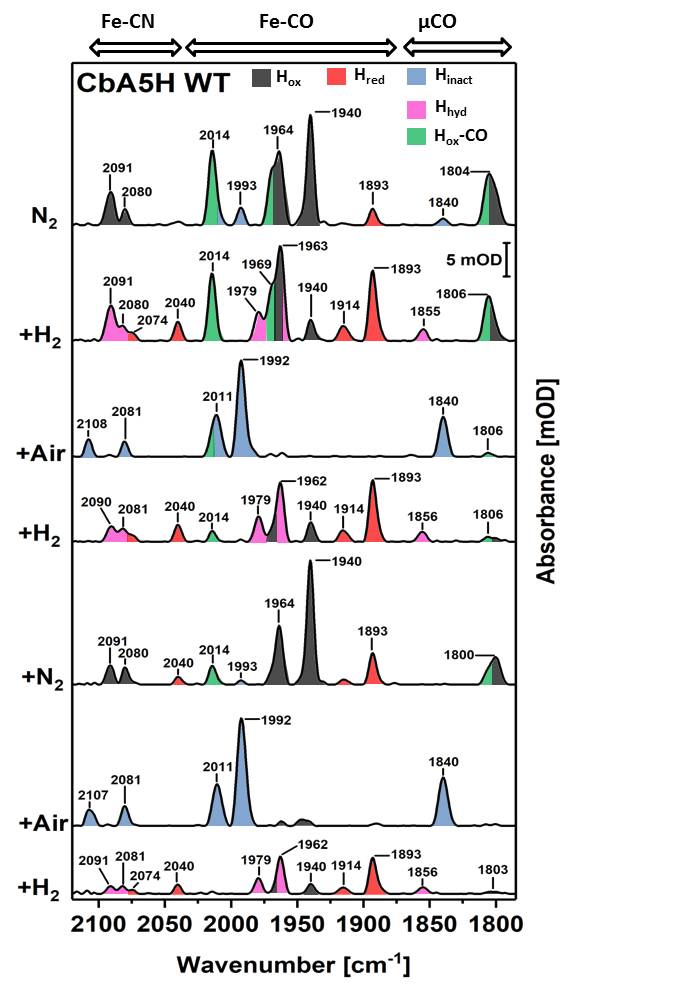


**Supplementary Fig. 1| IR-vibrational spectra of CbA5H^WT^ prior and during iterative cycles of O_2_ exposure, H_2_- and N_2_-purging monitored *via* ATR-FTIR-spectroscopy.** The same protein film (pH8) was used throughout all measurements and different gasses (H_2_, N_2_, air) were applied according to the order indicated in the spectra. Starting with N_2_, CbA5H is predominantly in the H_ox_ state with fractions of H_ox_-CO (probably due to CO release during *in vitro* maturation in which a 10-fold molar excess of the [2Fe]^MIM^ complex was used [^1^](#_ENREF_1)) as well as a minor fraction of H_inact_. Upon switching to H_2_ (gas mixture of 80% H_2_ and 20% N_2_), H_hyd_ and H_red_ are enriched after a few minutes while the H_ox_-CO fraction remains unaffected. Exposure to air (0.03 l∙min^-1^) leads to a quantitative accumulation of the H_inact_ state after 2 minutes. Switching back to H_2_, the catalytic states H_red_ and H_hyd_ reappear; remarkably, H_ox_-CO (maker band at 2014 cm^-1^) is now strongly decreased, demonstrating that the formation of the H_inact_ state leads to the release of the surplus CO-ligand as a consequence of bond-formation between Cys367 and Fe_d_, thereby replacing exogenous CO). Switching back to N_2_ gas, the H_ox_-state is enriched again. Subsequent cycles of air exposure and H_2_-purging demonstrates the full reversibility of the transition to the O_2_ protected H_inact_ state.


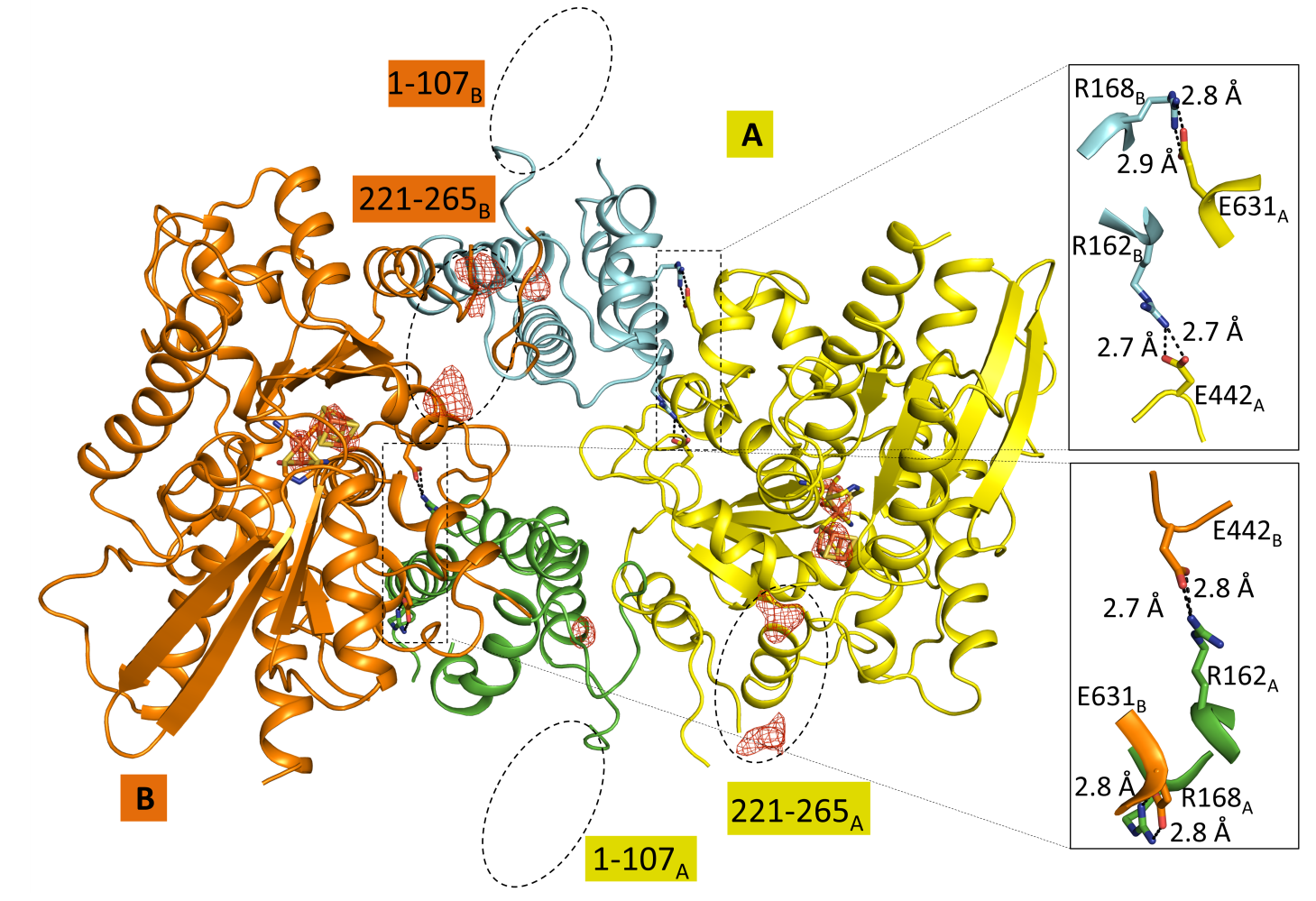


**Supplementary Fig. 2| Crystal structure of the CbA5H^air^ homodimer including monomers A and B.** The cartoon model of monomer A is colored in yellow as in Fig 2. In monomer B, the H- and F-domains are shown in orange and the SLBB domain in cyan. The anomalous density contoured at 4 σ is shown to indicate the localization of the iron-sulfur clusters. Four pairs of salt bridges may be important for dimerization: R168_B_-E631_A_, R162_B_-E442_A_, R168_A_-E631_B_ and R162_A_-E442_B_. The biological assembly as a dimer was confirmed *via* gel filtration.


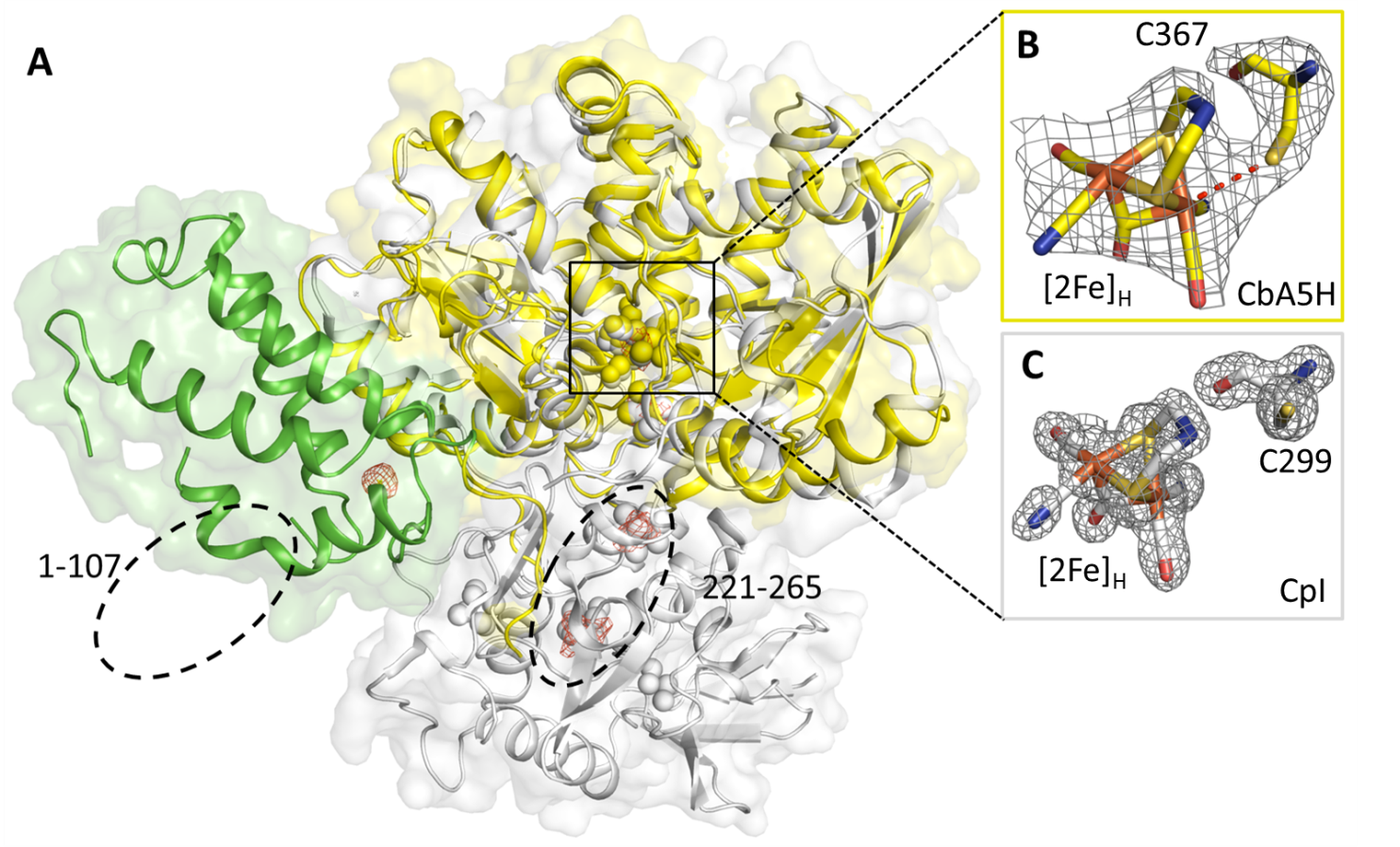


**Supplementary Fig. 3| Structural comparison of CbA5H^air^ monomer with CpI.** (**A**) Superimposition of CbA5H^air^ and CpI; CpI is colored in white, CbA5H is shown in yellow and green: the yellow part represents the well-conserved catalytic H-domain. Except for the additional cluster in the SLBB domain (green), the anomalous density (omitting map contoured at 4 σ) is well aligned with the corresponding iron-sulfur clusters in CpI. The stick models of the [2Fe]_H_ cluster and the conserved cysteine of the proton transfer pathway (CbA5H-C367/CpI-C299) are highlighted in (**B**) for CbA5H and (**C**) for CpI, respectively. Simulated annealing omitting maps were contoured at 2 and 4 σ for CbA5H and CpI, respectively.


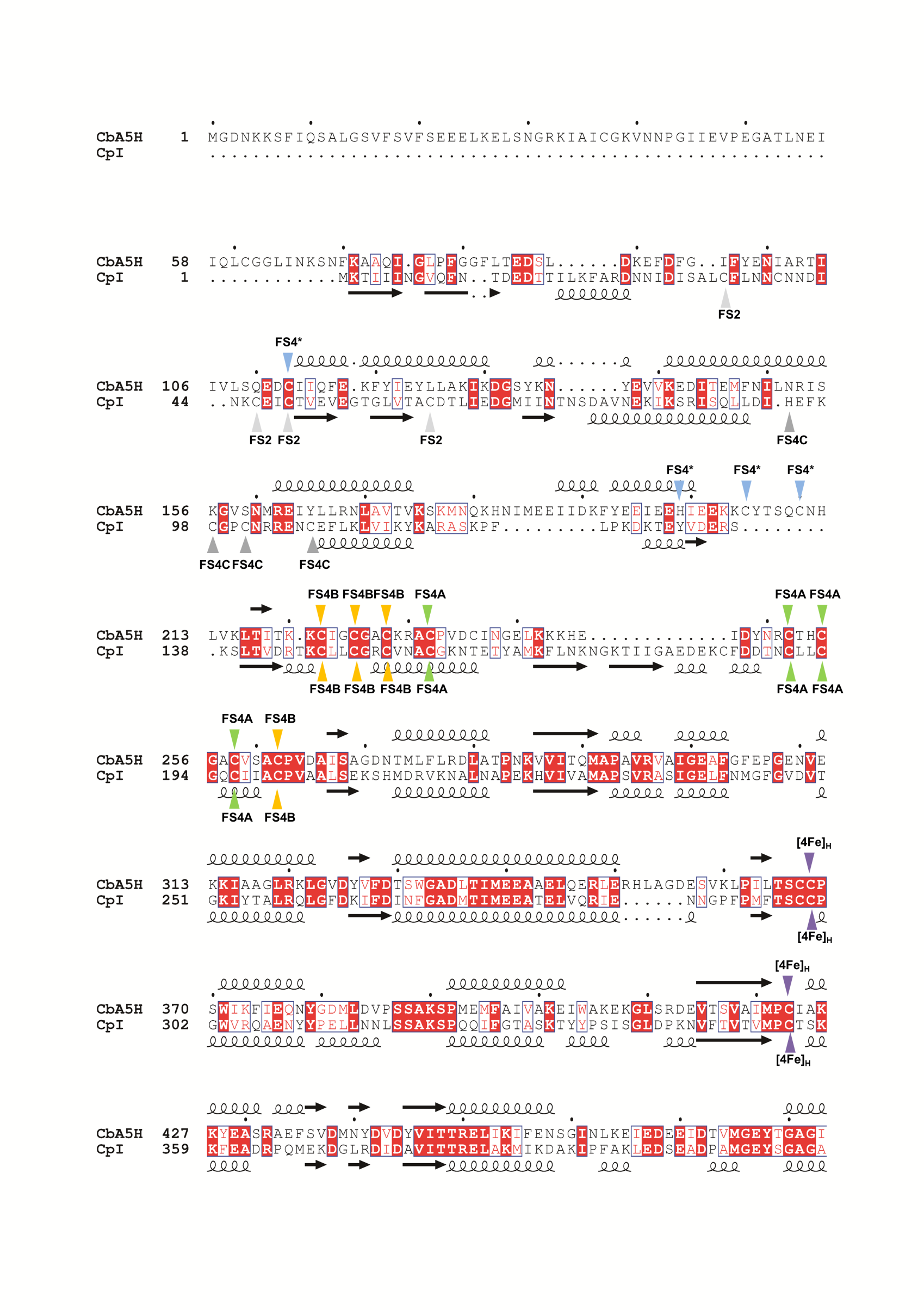


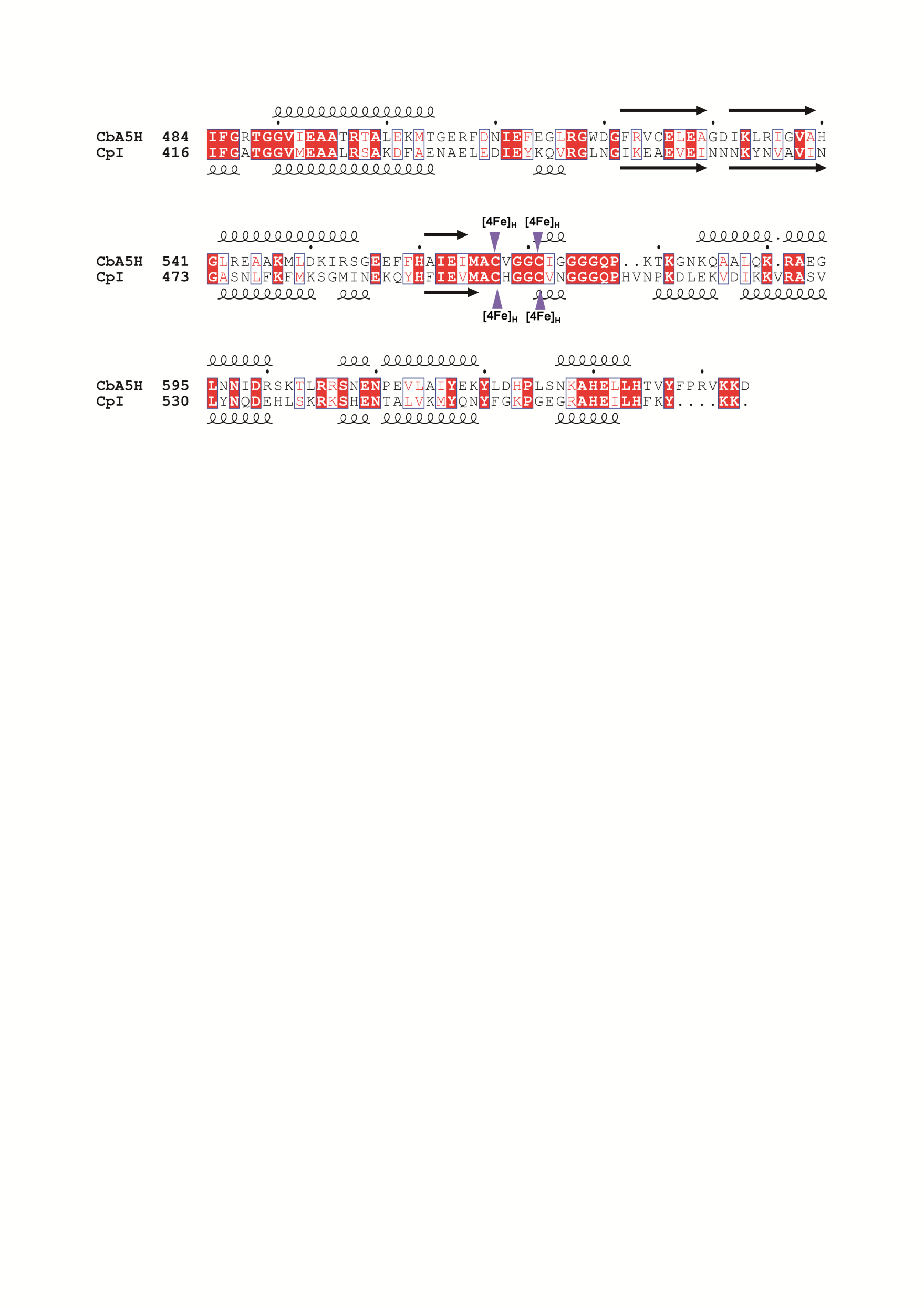


**Supplementary Fig. 4| Sequence alignment of CbA5H with CpI.** Residues indicated by a red background and white text are highly conserved, while residues depicted in red text are partially conserved. The coordinating residues (Cys or His) of individual iron-sulfur clusters are highlighted by triangles of the same color. The sequences alignment was done with ClustalW [^2^](#_ENREF_2) and plotted by using ESPript [^3^](#_ENREF_3). The putative coordinating residues for the presumptive [4Fe4S] cluster (FS4*) in the SLBB domain are C113, H199, C205 and C210. The sequence CX_n_HX_5_CX_4_C (X represents any amino acid) suggests a new type of iron-sulfur cluster motif of yet unknown function.


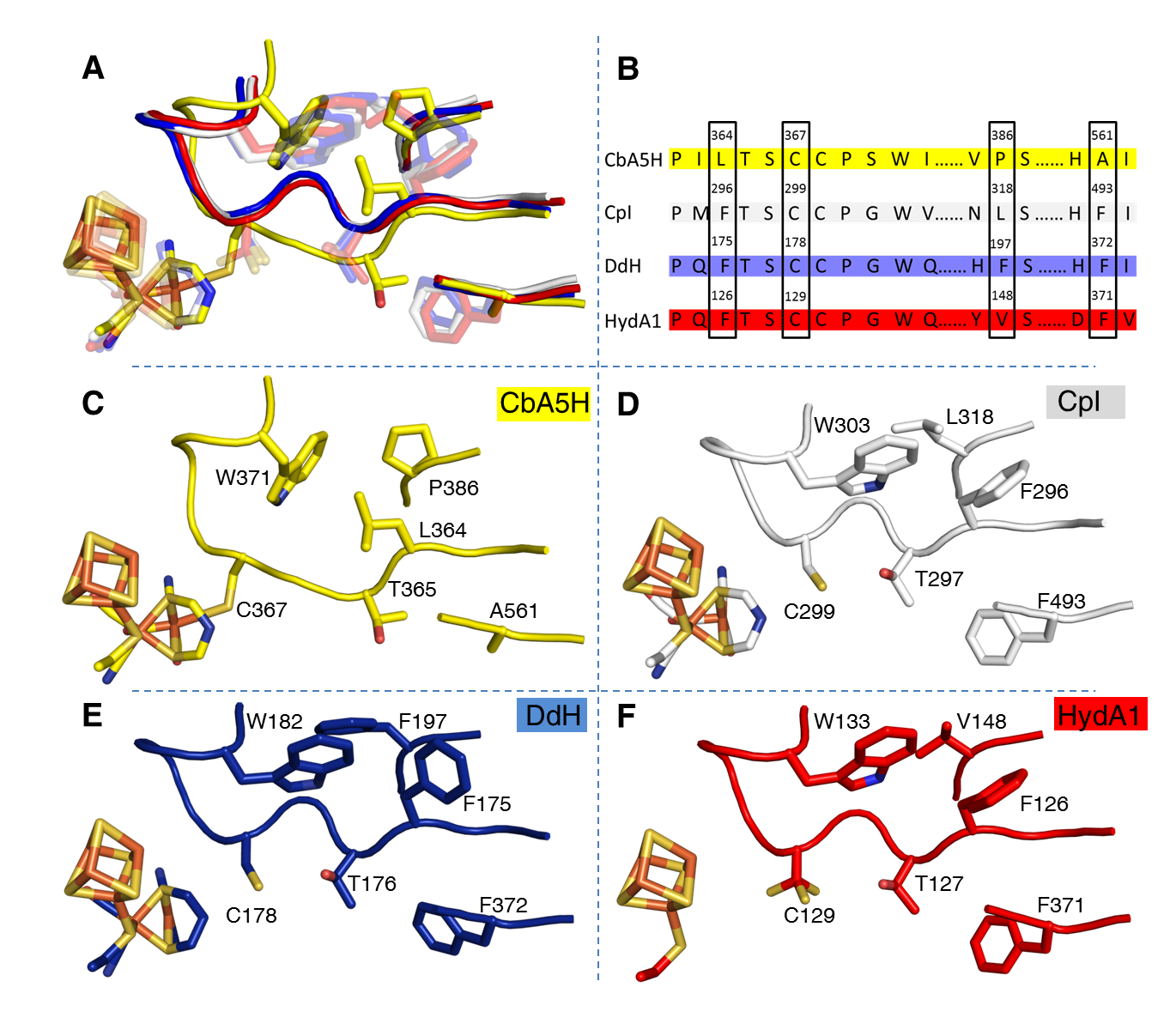


**Supplementary Fig. 5| Structure and sequence of the local environment of the flipped peptide loop in CbA5H^air^ compared to CpI, DdH and HydA1. (A)** Superposition of all structures. For clarity, CpI, DdH and HydA1 are shown half transparent. The structures are show individually in panels (**C)**, (**D)**, (**E)** and (**F)** for CbA5H^air^, CpI, DdH and HydA1, respectively. Panel (**B)** shows the corresponding sequence alignment for the loop region and positions in its local environment. The positions investigated in this study are highlighted by rectangles. The PDB IDs for CbA5H, CpI, DdH and HydA1 are 6TTL, 4XDC, 1HFE and 3LX4, respectively.


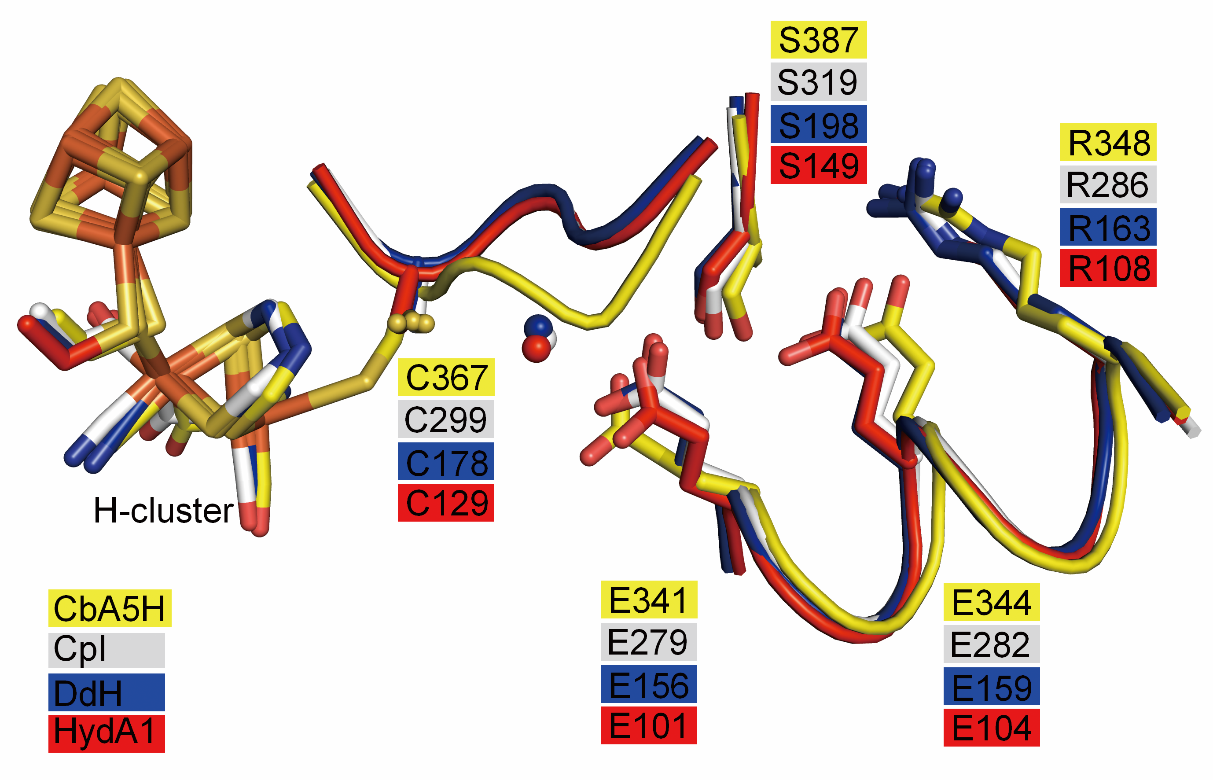


**Supplementary Fig. 6| Structural alignment of the residues in the proton transfer pathway of CbA5H^air^, CpI, DdH and HydA1.** Overall, the entire proton transfer pathway is structurally conserved in CbA5H^air^, besides the movement of C367. Due to the relatively low resolution of the CbA5H^air^ structure, the water molecules that are part of the pathway cannot be observed. The color code is the same as in Supplementary Figure 5. For clarity, the other two conformations of C129 in apo-HydA1 are omitted. The PDB IDs for CbA5H, CpI, DdH and HydA1 are 6TTL, 4XDC, 1HFE and 3LX4, respectively.

**Supplementary Fig. 7| H_2_ production activities of CpI, CbA5H^WT^ and site-directed mutagenesis variants.** Activities were measured with 100mM NaDT as sacrificial electron donor and 10mM MV as electron mediator. **(A)** Methyl viologen specific H_2_ production activities of all wildtype enzymes and SDM variants examined in the present study, measured at pH 6.8. **(B)** Comparison of the normalized pH dependent H_2_ production activity profiles (from 5 to 9) of CbA5H^WT^ and variant C367D. Data are presented as mean values ± SD, error bars indicate standard deviations (n=3). Source data are provided in a source data file. For panel a, individual values are indicated by red, green and blue markers.

**Supplementary Fig. 8|** **Anaerobic oxidative inhibition in cyclic voltammograms of CbA5H^WT^ and HydA1**. In case of HydA1 **(a)**, anaerobic oxidative inhibition starts at a significantly higher potential (close to 0V vs SHE; broken green line) and results from the potential-dependent interaction of chloride ions in the buffer (100 mM potassium phosphate buffer, 100 mM NaCl) with the H-cluster [^4^](#_ENREF_4). For CbA5H **(b)**, anaerobic oxidative inhibition already starts at -0.3V vs SHE (broken black line) and corresponds to the reversible formation of H_inact_. Both cyclic voltammograms were recorded under the same conditions (T = 10 °C, pH 7, 1 atm of H_2_, scan rate: 20 mV/s, electrode rotation rate: 1000 rpm). Source data are provided in a source data file. For each enzyme the experiment was performed at least three times, with similar results.

**Supplementary Fig. 9| Influence of NaCl concentration on anaerobic oxidative inhibition.** Normalized cyclic voltammograms of CbA5H^WT^ (black/grey; scan rate:3 mV/s) and CbA5H variant C367D (different shades of blue; scan rate: 10 mV/s) recorded at pH 7 and 5°C in 0.1M phosphate buffer with increasing NaCl concentration (in the range of 0 to 1.4 M). Source data are provided in a source data file.

**Supplementary Fig. 10| Redox state fractions of CbA5H variants C367D and C367A prior and during O_2_ exposure monitored *via* ATR-FTIR-spectroscopy**. Experimental conditions are the same as those for the CbA5H^WT^ experiment in Supplementary Figure 1. IR spectroscopy experiments were performed independently at least two times with similar results. Source data are provided in a source data file.

**
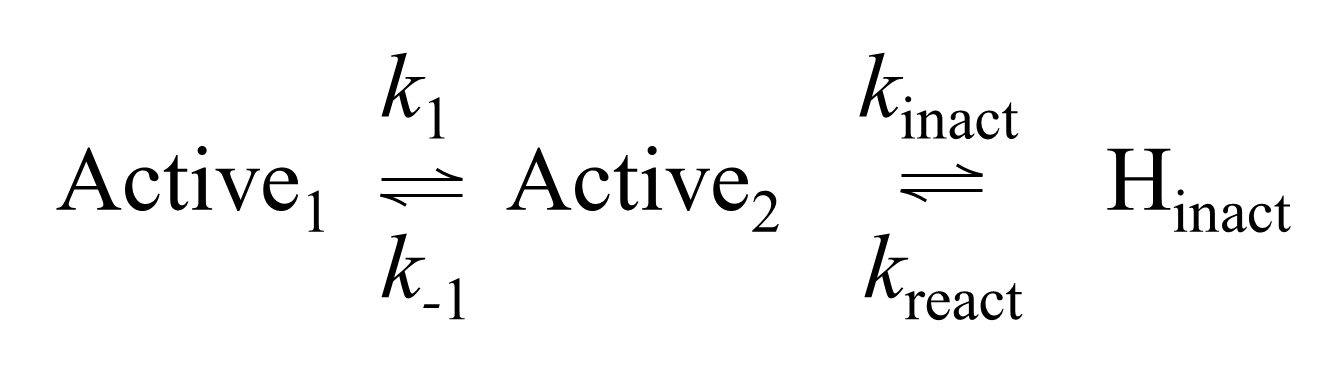
**

**Supplementary Fig. 11| Plot of the rate constants that define the AAI model (Active 1↔Active 2↔H_inact_) against potential (E) at different pH values**, based on the analyses of chronoamperograms recorded for CbA5H^WT^ at pH 10 (squares), 8.5 (triangle) and 7 (circle). Only *k*_react_ is significantly dependent on pH and electrode potential.

**Supplementary Fig. 12|** **Plot of the ratio I_A1_/I_A2_ against electrode potential obtained from fitting the AAI model to the chronoamperometric traces of CbA5H^WT^ and the four variants.**

**Supplementary Fig. 13| Redox state fractions of loop region variants A561F, L364F, P386L and L364F-A561F of CbA5H prior and during O_2_ exposure, monitored *via* ATR-FTIR-spectroscopy**. Experimental conditions correspond to the measurement of CbA5H^WT^ as described in Supplementary Figure 1. IR spectroscopy experiments were performed independently at least two times with similar results. Source data are provided in a source data file.

**Supplementary Fig. 14**| **Plot of rate constant *k*_react_ against potential (E) at pH 7, based on the analyses of chronoamperograms recorded for CbA5H^WT^ with the AAI model.**

**Supplementary Fig. 15|** **H-bond contacts stabilizing the states A1 and H_inact_ in CbA5H. (a)** Putative H-bond network stabilizing the configuration of the TSC loop in CpI which likely corresponds to the A_1_ state of CbA5H. Water molecules contributing to the H-bond network are depicted as green balls. (**b)** H-bond network stabilizing the rotated TSC loop configuration, initiating the shift of W371 and of the corresponding alpha helix which leads to the translocation of C367. Unfortunately, the moderate resolution of CbA5H^air^ does not permit the localization of the positions of contributing water molecules in the H_inact_ state of CbA5H^air^. As the water molecules depicted in panel A are conserved in the structures of FeFe hydrogenases one can only speculate about their position according to available space and potential H-bond partners (broken circles and parentheses and).

**Supplementary Fig. 16|** **Comparison of local b-factor differences in the x-ray structures of CpI (pdb code: 4xdc) and CbA5H^air^ (6TTL)**. **a-b:** Cartoon structure models of CpI and CbA5H^air^ colored according to the b-factor of individual structure parts. **c-d:** Close-up of the H-cluster and the TSC loop region with residues participating in the O_2_-resistance mechanism depicted as stick structures. No significant b-factor deviation is visible in the general environment of the H-cluster or the TSC-loop region. The dominating blue color range indicates for both structures likewise low b-factors in the center of the H-domain.

**
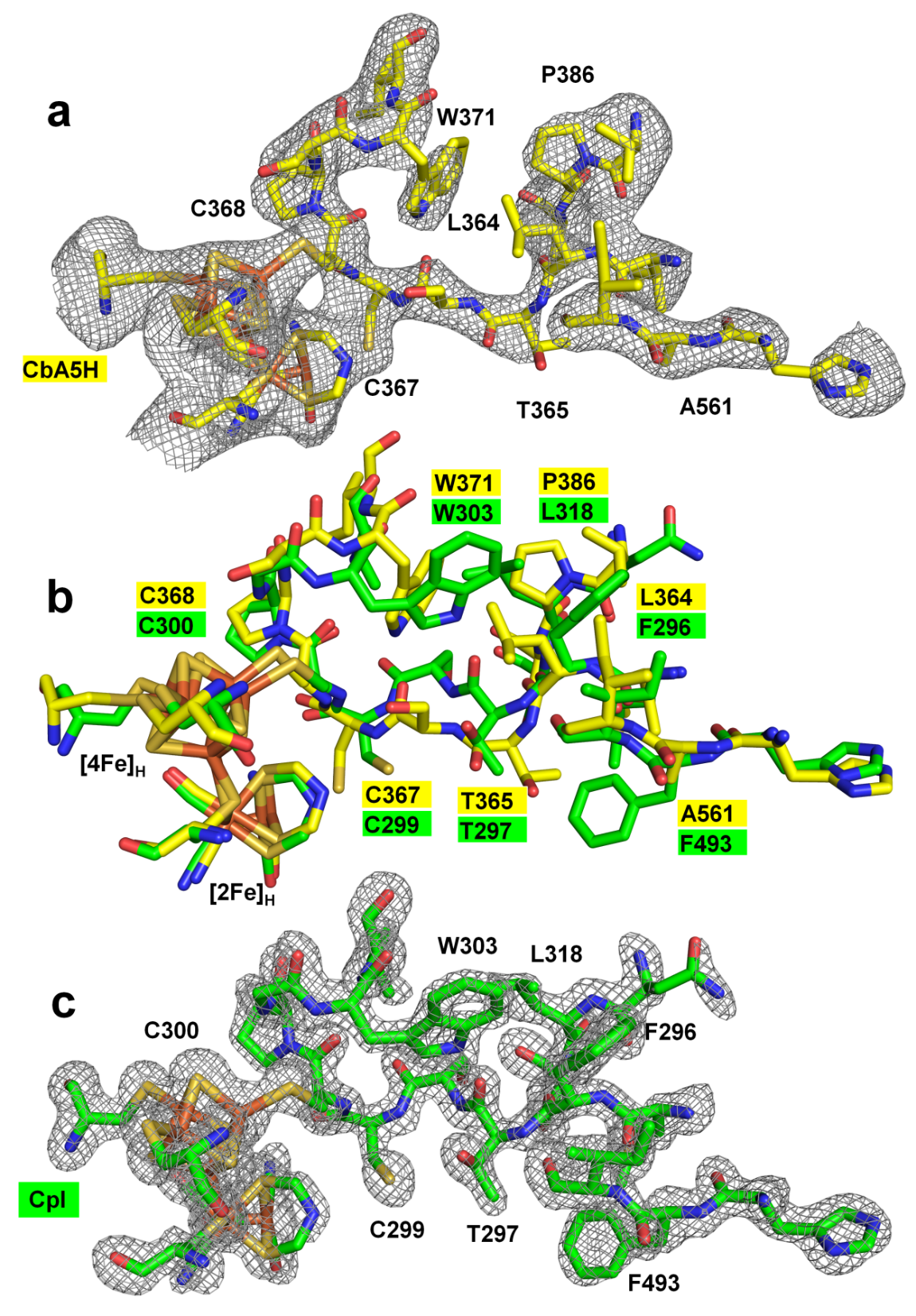
**

**Supplementary Fig. 17|** **Structural comparison between CbA5H and CpI.** Panels **a** and **c** show simulated omit maps (Fo-Fc) for CbA5Hair and CpI contoured at 2 and 3 σ, respectively over the corresponding stick structures. Panel **b** depicts a superposition of stick models for CbA5Hair and CpI, including the H-cluster, the loop region and its local environment. Carbon atoms were colored in yellow and green for CbA5Hair and CpI respectively.

**Supplementary Tables:**

**Supplementary Table 1|** **Statistics of data collection for CbA5H^air^ (PDB-ID: 6TTL).**

| **Data collection** | **Excluding anomalous scattering** | **Including anomalous scattering** |
| --- | --- | --- |
| X-ray source | ESRF ID29 | |
| Wavelength (Å) | 0.976 | |
| Space group^a^ | P 4_2_ 2_1_ 2 | |
| **Cell dimensions** | | |
| a, b, c (Å) | 169, 169, 127 | |
| α, β, γ (°) | 90.00, 90, 90.00 | |
| Resolution (Å) | 49.26-2.9 (3.0-2.9)^b^ | 20.31-2.96 (3.065-2.96)^b^ |
| *R*_merge_ | 0.2203 (2.628) | 0.209 (2.132) |
| I / σ(I) | 13.36 (1.14) | 10.47 (1.06) |
| Completeness (%) | 99.78 (99.95) | 99.53 (99.92) |
| Redundancy | 26.4 (25.6) | 14.1 (14.0) |
| CC1/2 | 0.999 (0.517) | 0.999 (0.452) |
| Unique reflections | 41295 (4063) | 73030 (7320) |

a As a control, data were also processed in space group P1 and all relevant tetragonal space groups. In

these cases, either molecular replacement failed, or crystallographic statistics and map qualities were

clearly inferior to space group P42 21 2.’

b Numbers in brackets indicate values in the highest resolution shell.

**Supplementary Table 2|** **Statistics of structure refinement for CbA5H^air^ (PDB-ID: 6TTL).**

| **Refinement** | |
| --- | --- |
| Resolution (Å) | 49.26-2.9 |
| No. reflections | 41225 |
| *R*_work_ / *R*_free_ | 0.3385 / 0.3572 |
| **No. atoms** | |
| Protein | 7598 |
| Ligand | 50 |
| **B-factors** | |
| Wilson B factor | 87.5 |
| Protein | 71.74 |
| Ligand | 62.42 |
| **R.m.s deviations** | |
| Bond lengths (Å) | 0.004 |
| Bond angles (°) | 0.9 |
| **Ramachandran statistics** | |
| Favored region (%) | 91.72 |
| Allowed region (%) | 7.86 |
| Outliers (%) | 0.42 |
| **Occupancy (for ligand)** | |
| [4Fe]_H_ (A/B)^a^ | 0.89/0.90 |
| [2Fe]_H_ (A/B) | 1.00/1.00 |

a A/B represents values in two monomers chain A and chain B respectively.

**Supplementary Table 3| Primers used in QuikChange PCR to generate expression constructs for site-directed mutagenesis variants of CbA5H.**

|  | **Forward primer** | **Reverse primer** |
| --- | --- | --- |
| **L364F** | CTGCCGATTTTCACCAGCTGCTGCCCGAG | CTGGTGAAAATCGGCAGTTTCACGCTTTCATC |
| **C367A** | CCAGCGCGTGCCCGAGCTGG | GGGCACGCGCTGGTCAGAATCGG |
| **C367D** | CCAGCGATTGCCCGAGCTGG | GGGCAATCGCTGGTCAGAATCGG |
| **P368L** | GATGTGCTGAGCAGCGCGAAAAGCC | CGCTGCTCAGCACATCCAGCATATCG |
| **A561F** | GAATTTTTTCATTTTATTGAAATTATGGCGTGCGTGGG | CATAATTTCAATAAAATGAAAAAATTCTTCGCCGCTGCGAATTTTATC |

**Supplementary notes**

1. **Structure determination**

Despite intensive modelling attempts, the first 107 amino acids of the N-terminus and the bacterial ferredoxin domain (residues 221-265 coordinating two [4Fe4S] clusters (FS4A and FS4B); the eight ligation cysteines are all conserved, see Supplementary Figures 3 and 4)[^5^](#_ENREF_5) had to be omitted from the final model. Although significant electron density was observed for both domains, modelling was unsuccessful. The large B factors (200-400) for the two [4Fe4S] clusters in the ferredoxin domain indicate a significant disorder. However, the anomalous density undoubtedly shows their positions which align well with the clusters in CpI (Supplementary Figure 3).

As for the N-terminal domain, the electron density for a β-sheet with several β-strands is present, consistent with the assignment of an SLBB domain. Placement of a main chain model in these β-strands decreases the R_free_/R_work_ by 2-3%. However, the phase errors, FOMs (figure of merit) and overall density did not improve significantly upon modelling the backbone of the SLBB domain. The electron density of the sidechains does not allow the assignment of the sequence, underlining the inherent flexibility of the domain.

To further explore the possibilities of modelling the SLBB domain, two different strategies were followed. To avoid potential disorder due to cryocooling, we collected X-ray data at room temperature (RT). Several full datasets collected at RT indeed improved the disorder significantly (Wilson B factors 55 vs. 87.5, Supplementary Table 1). To achieve a full dataset at the highest possible resolution of 2.9 Å (same as the one presented in the manuscript) with RT data, we merged the data from 5-10 crystals. Nevertheless, it was not possible to model the N-terminal part of the SLBB domain in the resulting maps despite improved density in the H-domain. The best resolution gained from a single crystal measured at room temperature was 3.2 Å due to severe radiation damage.

To resolve the missing part of the SLBB domain that contains two cysteine residues, we collected further data sets with high redundancy at longer wavelength (2.0 Å) to make use of the anomalous signal of sulfur atoms for localization and for phase improvement. However, these data did not contain enough information for further modelling, as even the anomalous densities of sulfur atoms in the well-resolved H-domain were not very defined.

Anomalous density in the SLBB domain is surrounded by the sidechains of C113, H199, C205 and C210 which could serve as FeS-cluster ligands. The ligand stoichiometry strongly suggesting the presence of a [4Fe4S]-cluster. When modelled and refined, an occupancy of ~40% and unsatisfactory ligation geometry indicated a partial degradation of the cluster. Modelling of a [2Fe2S]-cluster was also attempted and resulted in full occupancy. However, a ligand assignment for a typical [2Fe2S]-cluster with each Fe atom being coordinated either by 2 cysteines or by e.g. one cysteine and one histidine, was not possible. The cluster was also omitted in the final model, even though we consider it likely that it is a [4Fe4S]-cluster.

Overall, the combination of limited resolution, presence of tNCS (translational non-crystallographic symmetry) and flexible domains absent in the model explains the somewhat unsatisfactory statistics in Table S1. One needs to stress, that all molecular features discussed in the manuscript are present in the corresponding simulated annealing omit maps or in anomalous maps.

After deposition of the structure, we were able to collect a dataset at the iron K edge wavelength for a different crystal form (not presented in the manuscript) with similar resolution of around 3 Å. Experimental phasing was successful by running AutoSol (SAD method) with an FOM of 0.459[^6^](#_ENREF_6). The iron sites confirm the cluster positions discussed in the text. An automatically build model based on these experimental phases alone (PHENIX AutoBuild wizard [^7^](#_ENREF_7)) superposes well with 6TTL (chain B) with a root mean square deviation of 0.4 Å for 296 C-α atoms (pymol align). This strengthens the structural model phased by molecular replacement presented in this work.

1. **Kinetic modelling of anaerobic inactivation of CbA5H**

We fitted several kinetic models to the chronoamperometric traces. For each model, we assumed that:

- the current is the sum of the currents of each species weighted by the fractions of the corresponding species;
- the rate constants are independent of time and, in a single experiment, only depend on potential (i.e., all the potential steps at the same potential have the same value of the rate constants);
- potential steps have no instant effect on the concentrations of the species;
- the concentration of the inactive states is 0 at the beginning of the first step;
- film loss can be represented by a potential-dependent first-order rate constant that is the same for all species.

These hypotheses are the same as those we used in our previous works [^8^](#_ENREF_8). Fitting a single experiment yields the values of the rate constants at the two potentials used in this experiment. The quality of each model was assessed first by the quality of the fits (residuals), and then by the quality of the resulting rate constants: reproducibility of the values of the rate constants at a given potential for different experiments, continuity of the rate constants as a function of potential [^9^](#_ENREF_9).

For each fit, to make sure that the fitting procedure finds the best parameters, we started 150 different fits with random initial parameters and kept only the best final set of parameters (this was made possible using the new “Monte-Carlo” parameter space explorer available in version 3.0 of QSoas). We used fit engines based on the ODRPACK code [^10^](#_ENREF_10).

We first tested a simple two-species model like the following:

1. ${Active}_{\overset{\leftarrow}{k_{react}}}^{\underset{\to}{k_{inact}}} Inactive$

The fits of this model were very unsatisfactory, since it only predicts mono-exponential evolutions over time, while the experimental traces are clearly biphasic. We then investigated two models that include 3 species:

1. ${Active 1}_{\overset{\leftarrow}{{kreact}}}^{\underset{\to}{k_{inact}}} {Inactive 1}_{\overset{\leftarrow}{k_{-1}}}^{\underset{\to}{k_{1}}} Inactive 2$

(named AII) and

1. ${Active 1}_{\overset{\leftarrow}{k_{-1}}}^{\underset{\to}{k_{1}}} {Active 2}_{\overset{\leftarrow}{k_{react}}}^{\underset{\to}{k_{inact}}} Inactive$

(named AAI).

In each model, we adjusted the following parameters for each potential: *k*_inact_, *k*_react_, *k*_1_, *k*_-1_, *k*_loss_ (film loss) and a current for the active species. For the AAI model, the initial ratio [A_1_]/[A_2_] was also adjusted, and in this first step, we assumed that both A_1_ and A_2_ have the same activity. While both the AII and AAI gave equally good fits when looking at the residuals, the rate constants given by the AII model were not continuous and inconsistent across different experiments, and additionally the best parameters gave systematically *k*_1_=*k*_-1_. On the other hand, the rate constants given by the AAI fits were continuous and consistent across potentials. In a second step, since we observed that the values of *k*_1_, *k*_-1_ and *k*_inact_ varied only slightly as a function of potential (see Supplementary Figure 11), we fitted the AAI mode simultaneously to all the chronoamperometric traces recorded at a given pH, assuming that *k*_1_, *k*_-1_ and *k*_inact_ were constant. The constraints added were sufficient to allow the independent determination of the currents of the A_1_ and A_2_ species (i.e. for these fits, we did not assume that they were identical, Supplementary Figure 12 shows the ratio of the two activities plotted against electrode potential: it is essentially constant around I_A1_/I_A2_=2). We estimated the errors on the fit parameters by comparing the final values of several fits giving similar residuals.

By varying the values of the potential steps and the pH and repeating the analysis, we derived the dependence of the four rate constants on pH and potential shown in Figure 4B. We initially observed that the values of the rate constants *k*_1_, *k*_-1_ and *k*_inact_ vary very little over a large range of potential (Supplementary Fig. 11) and subsequently used this information to constrain the fitting procedures and reduce the number of adjustable parameters (hence the absence of scatter for the values of these three parameters in Figure 4B).

**Supplementary Discussion**

**Supplementary Discussion 1**

The crystal structure of CbA5H^air^ was solved at a resolution of 2.9 Å, showing a homodimeric state (Supplementary Figure 2). In addition to sequence- and structure-alignments with CpI (an isoform of CpIII) and DdH (Supplementary Figs. 3-5), the anomalous density map supports the presence of an F-domain with two F-clusters (Figs 2A and Supplementary Figure 3) (Fig. 2B), which is a characteristic feature of M2-type [FeFe]-hydrogenases [^11^](#_ENREF_11)^,^ [^12^](#_ENREF_12). We confirm the presence of an additional protein domain (partially disordered in our structure) located N-terminal of the F-domain, which has been classified as a soluble ligand-binding beta-grasp (SLBB) domain (Fig. 2A) [^13^](#_ENREF_13)^,^ [^14^](#_ENREF_14). Here, the anomalous electron density suggests the presence of another [FeS]-cluster of unknown function (FS4* in Fig. 2B + Fig. 2C).

**Supplementary Discussion 2**

In CpI and HydA1 the replacement of this cysteine with alanine was shown to induce the accumulation of the so-called H_hyd_ state in the presence of H_2_ [^15^](#_ENREF_15)^,^ [^16^](#_ENREF_16). The C367A variant shows the same behaviour under H_2_, with characteristic IR-signals at 1978, 1962 and 1855 cm^1^, remaining stable even after prolonged exposure to N_2_ (Supplementary Figure 10). Upon exposure to air, the IR-signals of the C367A variant are quickly replaced with the characteristic spectrum of the “H_ox_-O_2_” state, previously characterized for the corresponding HydA1 variant C169A [^17^](#_ENREF_17). The cysteine to alanine exchange impedes proton transfer and leads to the accumulation of the primary O_2_-adduct H_ox_-O_2_, preventing the formation of ROS and thus H-cluster degradation. Observing the O_2_-bound state in C367A demonstrates that the resistance of CbA5H to O_2_ is not due to a specific feature of the enzyme which limits O_2_ diffusion to the H-cluster. Instead it is clearly dependent on the presence of C367.

**Supplementary References**

1. Esselborn J*, et al.* Spontaneous activation of [FeFe]-hydrogenases by an inorganic [2Fe] active site mimic. *Nat Chem Biol* **9**, 607-609 (2013).

2. Thompson JD, Higgins DG, Gibson TJ. CLUSTAL W: improving the sensitivity of progressive multiple sequence alignment through sequence weighting, position-specific gap penalties and weight matrix choice. *Nucleic acids research* **22**, 4673-4680 (1994).

3. Robert X, Gouet P. Deciphering key features in protein structures with the new ENDscript server. *Nucleic acids research* **42**, W320-324 (2014).

4. Del Barrio M*, et al.* Interaction of the H-Cluster of FeFe Hydrogenase with Halides. *J Am Chem Soc* **140**, 5485-5492 (2018).

5. Peters JW*, et al.* [FeFe]- and [NiFe]-hydrogenase diversity, mechanism, and maturation. *Biochimica et biophysica acta* **1853**, 1350-1369 (2015).

6. Terwilliger TC*, et al.* Decision-making in structure solution using Bayesian estimates of map quality: the PHENIX AutoSol wizard. *Acta crystallographica Section D, Biological crystallography* **65**, 582-601 (2009).

7. Terwilliger TC*, et al.* Iterative model building, structure refinement and density modification with the PHENIX AutoBuild wizard. *Acta crystallographica Section D, Biological crystallography* **64**, 61-69 (2008).

8. Fourmond V, Infossi P, Giudici-Orticoni MT, Bertrand P, Leger C. "Two-step" chronoamperometric method for studying the anaerobic inactivation of an oxygen tolerant NiFe hydrogenase. *J Am Chem Soc* **132**, 4848-4857 (2010).

9. Fourmond V*, et al.* SOAS: a free program to analyze electrochemical data and other one-dimensional signals. *Bioelectrochemistry* **76**, 141-147 (2009).

10. Boggs PT, Donaldson JR, Byrd RH, Schnabel RB. Algorithm 676: ODRPACK: software for weighted orthogonal distance regression. *ACM Trans Math Softw* **14**, 348-364 (1989).

11. Meyer J. [FeFe] hydrogenases and their evolution: a genomic perspective. *Cell Mol Life Sci* **64**, 1063-1084 (2007).

12. Nicolet Y, Piras C, Legrand P, Hatchikian CE, Fontecilla-Camps JC. Desulfovibrio desulfuricans iron hydrogenase: the structure shows unusual coordination to an active site Fe binuclear center. *Structure* **7**, 13-23 (1999).

13. Morra S, Arizzi M, Valetti F, Gilardi G. Oxygen Stability in the New [FeFe]-Hydrogenase from Clostridium beijerinckii SM10 (CbA5H). *Biochemistry* **55**, 5897-5900 (2016).

14. Burroughs AM, Balaji S, Iyer LM, Aravind L. A novel superfamily containing the beta-grasp fold involved in binding diverse soluble ligands. *Biology direct* **2**, 4 (2007).

15. Duan J*, et al.* Crystallographic and spectroscopic assignment of the proton transfer pathway in [FeFe]-hydrogenases. *Nature communications* **9**, 4726 (2018).

16. Winkler M*, et al.* Accumulating the hydride state in the catalytic cycle of [FeFe]-hydrogenases. *Nature communications* **8**, 16115 (2017).

17. Mebs S*, et al.* Hydrogen and oxygen trapping at the H-cluster of [FeFe]-hydrogenase revealed by site-selective spectroscopy and QM/MM calculations. *Biochimica et biophysica acta* **1859**, 28-41 (2018).
