# Supplementary material for: A safety cap protects hydrogenase from oxygen attack
Source: Nat Commun. 2021 Feb 2;12:756. doi: 10.1038/s41467-020-20861-2 (PMC7854748; doi:10.1038/s41467-020-20861-2)
Supplement: Supplementary file 3 — Reporting Summary [file 41467_2020_20861_MOESM3_ESM.pdf]

nature research

Corresponding author(s): Thomas Happe, Christophe Léger

Last updated by author(s): Dec 7, 2020

Reporting Summary

Nature Research wishes to improve the reproducibility of the work that we publish. This form provides structure for consistency and transparency in reporting. For further information on Nature Research policies, see our Editorial Policies and the Editorial Policy Checklist.

Statistics

For all statistical analyses, confirm that the following items are present in the figure legend, table legend, main text, or Methods section.

n/a	Confirmed	
☐	☒	The exact sample size (n) for each experimental group/condition, given as a discrete number and unit of measurement
☐	☒	A statement on whether measurements were taken from distinct samples or whether the same sample was measured repeatedly
☒	☐	The statistical test(s) used AND whether they are one- or two-sided<br>*Only common tests should be described solely by name; describe more complex techniques in the Methods section.*
☒	☐	A description of all covariates tested
☒	☐	A description of any assumptions or corrections, such as tests of normality and adjustment for multiple comparisons
☐	☒	A full description of the statistical parameters including central tendency (e.g. means) or other basic estimates (e.g. regression coefficient) AND variation (e.g. standard deviation) or associated estimates of uncertainty (e.g. confidence intervals)
☒	☐	For null hypothesis testing, the test statistic (e.g. F, t, r) with confidence intervals, effect sizes, degrees of freedom and P value noted<br>*Give P values as exact values whenever suitable.*
☒	☐	For Bayesian analysis, information on the choice of priors and Markov chain Monte Carlo settings
☒	☐	For hierarchical and complex designs, identification of the appropriate level for tests and full reporting of outcomes
☒	☐	Estimates of effect sizes (e.g. Cohen's d, Pearson's r), indicating how they were calculated

Our web collection on statistics for biologists contains articles on many of the points above.

Software and code

Policy information about availability of computer code

Data collection	GC Solution (Shimadzu 2010) for H2 production data collection; OPUS (version 7.5, Bruker) for IR data collection; MXCuBE2 for crystallographic data collection in ESRF; PSTrace (version 5.2, PalmSens) for electrochemistry data collection.
Data analysis	We used Clone Manager Professional (Version 9) for the planning of cloning steps, designing primer sequences for site directed mutagenesis and verifying DNA sequences. For structural data processing, we used the following programs: XDS and XSCALE from the XDS Program Package (version March 15, 2019) and PHENIX 1.17.1_3660. Molecular replacement was carried out in Phaser (included in the Phenix installation). PyMOL (version 2.1.0) was employed to view and present structural models and electron densities. IR data, electrochemistry data and enzyme activity data were analyzed by using Origin (OriginPro 2019 (64-bit) 9.6.0.172).

For manuscripts utilizing custom algorithms or software that are central to the research but not yet described in published literature, software must be made available to editors and reviewers. We strongly encourage code deposition in a community repository (e.g. GitHub). See the Nature Research guidelines for submitting code & software for further information.

Data

Policy information about availability of data

All manuscripts must include a data availability statement. This statement should provide the following information, where applicable:

- Accession codes, unique identifiers, or web links for publicly available datasets
- A list of figures that have associated raw data
- A description of any restrictions on data availability

The coordinates and structure factors of CbA5Hair have been deposited in protein data bank (PDB) under PDB-ID: 6TTL [https://www.rcsb.org/structure/6TTL]. All data are available in the main text or the supplementary materials. Source data are provided in a source data file. Further data supporting findings of this study are available from the corresponding authors upon reasonable request. Further publicly available datasets used or indicated in this study comprise PDB-ID: 4XDC

[https://www.rcsb.org/structure/4XDC], PDB-ID: 3LX4 [https://www.rcsb.org/structure/3LX4] and PDB-ID:1HFE [https://www.rcsb.org/structure/1HFE].

Field-specific reporting

Please select the one below that is the best fit for your research. If you are not sure, read the appropriate sections before making your selection.

☒ Life sciences ☐ Behavioural & social sciences ☐ Ecological, evolutionary & environmental sciences

For a reference copy of the document with all sections, see nature.com/documents/nr-reporting-summary-flat.pdf

Life sciences study design

All studies must disclose on these points even when the disclosure is negative.

Sample size	For the structural data of Cba5Hair, we obtained 2 independent datasets with corresponding structural characteristics. From a comparison to our reference structure of CpI (pdb-ID: 4XDC) we located 4 polypeptide positions with potential impact on the characteristic O2 resistance of CbA5H and used these positions as target sites for site directed mutagenesis in CbA5H. We mostly followed the rational mutagenesis approach of substituting these positions with amino acids found at corresponding positions in CpI. We systematically characterized the resulting four single exchange variants and one double exchange variant in comparison to CbA5H wild type. We collected the data from at least three independent measurements for the in vitro hydrogen production assays (including the pH dependency experiment). IR spectroscopy experiments were done twice and the electrochemistry measurements for each wild type and variant protein were performed at least 3 times in independent experiments.
Data exclusions	No data were excluded
Replication	In solution activity assays, electrochemical examinations of individual wild type enzymes and mutagenesis variants were performed independently at least n=3 times with similar results. Bar diagrams depict mean values +/- SD including makers for individual values. IR spectroscopic measurements were done 2 times showing corresponding spectra.
Randomization	Randomization is not applicable because there were no experimental groups designated in this study.
Blinding	Blinding was not performed because it was not applicable to this study. This study was not dependent on the presence / absence of certain characteristics.

Reporting for specific materials, systems and methods

We require information from authors about some types of materials, experimental systems and methods used in many studies. Here, indicate whether each material, system or method listed is relevant to your study. If you are not sure if a list item applies to your research, read the appropriate section before selecting a response.

Materials & experimental systems

n/a	Involved in the study	
☒	☐	Antibodies
☒	☐	Eukaryotic cell lines
☒	☐	Palaeontology and archaeology
☒	☐	Animals and other organisms
☒	☐	Human research participants
☒	☐	Clinical data
☒	☐	Dual use research of concern

Methods

n/a	Involved in the study	
☒	☐	ChIP-seq
☒	☐	Flow cytometry
☒	☐	MRI-based neuroimaging
